# Decreased Serum Adipose Triglyceride Lipase Level Is Associated With Renal Function Impairment in Patients With Type 2 Diabetes

**DOI:** 10.1155/jdr/9987648

**Published:** 2025-07-24

**Authors:** Ying Wang, Tongtong Liu, Nan Li, Tingting Zhao, Xiai Wu, Yanmei Wang, Xi Dong, Hailing Zhao, Weijing Liu, Ping Li

**Affiliations:** ^1^Institute of Medical Science, China–Japan Friendship Hospital, Beijing, China; ^2^Key Laboratory of Chinese Internal Medicine of Ministry of Education and Beijing, Dongzhimen Hospital Affiliated to Beijing University of Chinese Medicine, Beijing, China; ^3^Department of Nephrology, Guang'anmen Hospital, China Academy of Chinese Medical Sciences, Beijing, China

**Keywords:** biomarker, diabetic kidney disease, serum ATGL, Type 2 diabetes mellitus

## Abstract

**Background:** Diagnosing diabetic kidney disease (DKD) remains a significant challenge. Research has increasingly focused on kidney injury resulting from lipid metabolism disorders. Adipose triglyceride lipase (ATGL), a pivotal enzyme in lipolysis, is essential for maintaining lipid metabolism balance. The objective of this study was to assess whether serum ATGL could serve as an early biomarker for DKD.

**Methods:** The study divided 236 participants into four groups: healthy controls (*n* = 59), Type 2 diabetes mellitus (T2DM) with albumin-to-creatinine ratio (ACR) < 30 mg/g (*n* = 80), microalbuminuria (L-DKD) with ACR 30–300 mg/g (*n* = 41), and macroalbuminuria (H-DKD) with ACR ≥ 300 mg/g (*n* = 56). Relevant clinical data were collected, and serum levels of ATGL, kidney injury molecule-1 (KIM-1), and tumor necrosis factor-1 (TNFR-1) were measured. Various statistical analyses, including Spearman's correlation test, receiver operating characteristic curve analysis, multivariate logistic regression, and restricted cubic spline (RCS), were employed to assess the relationship between serum ATGL levels and renal function impairment in DKD.

**Results:** Serum ATGL levels were notably lower in the T2DM, L-DKD, and H-DKD groups compared to healthy controls. Positive correlations were found between serum ATGL levels and estimated glomerular filtration rates (eGFR), while negative correlations were observed with diabetes duration, hypertension history, hyperlipidemia, urine ACR (UACR), 24-h urine total protein (UTP), serum creatinine (SCr), blood urea nitrogen, uric acid, TNFR-1, and KIM-1/creatinine (KIM-1/Cr) levels (*p* < 0.05). The receiver operating characteristic curve analysis demonstrated that the diagnostic performance of ATGL, when combined with traditional clinical markers, can enhance sensitivity. When participants were grouped by serum ATGL quartiles, it was observed that higher ATGL levels corresponded with lower UACR, 24 h-UTP, SCr, and TNFR-1 levels and higher eGFR. The odds ratios for elevated UACR and 24 h-UTP decreased, and eGFR increased with higher ATGL quartiles. Both univariate and multivariate logistic regression analyses indicated that serum ATGL is a protective factor against DKD development, even after adjusting for potential confounders. RCS analysis indicated a nonlinear dose–response association between serum ATGL levels and renal function metrics, specifically UACR and eGFR, in patients with DKD.

**Conclusion:** Serum ATGL levels are linked to reduced renal function in T2DM patients. A decline in ATGL levels corresponded with a nonlinear rise in UACR and a drop in eGFR, suggesting that serum ATGL may serve as a potential biomarker for DKD development.

## 1. Background

The global incidence of diabetes mellitus (DM) is increasing at an alarming pace. Among the complications associated with DM, diabetic kidney disease (DKD) is the most common and severe, being a primary cause of end-stage renal disease (ESRD) [1, 2]. The mortality risk in DKD is significantly higher compared to that in chronic kidney disease (CKD) without DM or in DM without DKD [3]. By 2040, CKD is expected to rank as the fifth leading cause of death worldwide, with DKD being the major contributor to CKD [4]. DKD is one of the leading contributors to the global disease burden [5]. Renal biopsy remains the definitive method for diagnosing DKD [6, 7]. It is an invasive histological study and is therefore usually limited to patients with atypical renal manifestations [8, 9]. The most widely used clinical markers for assessing DKD are a reduced estimated glomerular filtration rate (eGFR) and elevated albuminuria [10–12]. The eGFR is used for functional assessment, and albuminuria assesses structural kidney damage [13]. However, these two parameters are not specific to DKD, as they can also occur in individuals with other types of renal damage [14]. In about one-third of patients, renal function begins to decline prior to the development of proteinuria [15]. Consequently, the development of noninvasive biomarkers is essential for improving DKD management and reducing its clinical impact.

Adipose triglyceride lipase (ATGL) is a crucial enzyme in lipid metabolism and serves as the primary rate-limiting factor in lipolysis [16]. ATGL hydrolyzes triglycerides (TGs) into free fatty acids (FFAs) and diacylglycerol and belongs to the patatin-like phospholipase domain family [16, 17]. This enzyme is highly expressed in adipose tissue and localized in lipid droplets (LDs), which are closely associated with obesity [18–21]. ATGL can degrade the intracellular lipid storage organelles, LDs, through cytosolic lipase decomposition and lipid autophagy [16]. Renal lipotoxicity has been reported [22–24]. In patients with DKD, there is notable accumulation of lipid deposits and LDs in the kidneys, which can impair renal function [25, 26]. Thus, reducing ectopic renal lipid deposition can significantly protect renal function [27]. In addition, ATGL-deficient mice exhibit albuminuria and reduced creatinine (Cr) clearance, accompanied by ectopic lipid deposition (ELD) in the kidney, with proximal tubular lipid vacuolization and renal tubulointerstitial fibrosis [28, 29]. Nevertheless, the link between serum ATGL levels and DKD remains uncertain.

In this study, we assessed serum ATGL levels in healthy controls, Type 2 DM (T2DM) patients, and DKD patients with both microalbuminuria (L-DKD) and macroalbuminuria (H-DKD), aiming to explore the relationship between serum ATGL and renal function in T2DM. Tumor necrosis factor-1 (TNFR-1) was found to correlate with renal prognosis in T2DM patients with normal albuminuria (hazard ratio, 4.2; 95% confidence interval, 1.8–9.6). Additionally, kidney injury molecule-1 (KIM-1), a biomarker for renal tubular damage, may facilitate the uptake of fatty acids by renal tubular cells, promoting the progression of DKD [30–32]. Given previous findings linking TNFR-1 and KIM-1 with DKD outcomes, these biomarkers were selected for the combined analysis.

## 2. Methods

### 2.1. Study Design and Participant Selection

This study involved the recruitment of 236 participants from China–Japan Friendship Hospital, Guang'anmen Hospital, and Dongzhimen Hospital, including 59 healthy controls, 80 patients with T2DM, and 97 with DKD. Healthy control participants had negative diagnostic test results for T2DM or DKD [33]. The diagnosis of T2DM was confirmed based on the American Diabetes Association guidelines, which include criteria such as glycated hemoglobin ≥ 6.5%, fasting plasma glucose ≥ 126 mg/dL, and/or random plasma glucose ≥ 200 mg/dL [34]. Renal injury in patients with T2DM was characterized by the presence of proteinuria (albumin-to-creatinine ratio [ACR] >30 mg/g) and/or an abnormal eGFR of less than 60 mL/min/1.73 m^2^ [35]. Participants with an ACR of less than 300 mg/g were assigned to the L-DKD group, whereas those with an ACR of 300 mg/g or greater were categorized into the H-DKD group. During screening, we considered the onset timing of CKD patients with DM to facilitate the diagnosis of DKD or nondiabetic kidney disease (NDKD). NDKD is most commonly indicated by renal dysfunction occurring before the onset of diabetes, whereas DKD generally develops several years after diabetes onset [3]. We further excluded patients with NDKD as previously described [36]. The ethics committees of China–Japan Friendship Hospital (Approval No: 2023-KY-297), the lead institution for the clinical study, granted approval for this research. Additionally, written informed consent was obtained from all participants involved in the study.

### 2.2. Collection of Clinical Data

We gathered demographic and clinical information from all participants, along with routine biochemical tests. These included details such as age, sex, height, weight, duration of diabetes, and medical histories of hypertension, hyperlipidemia, fatty liver disease, cardiovascular conditions, cerebrovascular diseases, and smoking. Additionally, we measured urinary albumin-to-creatinine ratio (UACR), 24-h urine total protein (24 h-UTP), serum creatinine (SCr), blood urea nitrogen (BUN), serum uric acid (UA), homocysteine (HCY), total cholesterol (TC), TGs, high-density lipoprotein cholesterol (HDL-C), and low-density lipoprotein cholesterol (LDL-C) levels. Body mass index (BMI) was calculated by dividing weight (kilograms) by the square of height (square meters), while the eGFR was assessed using the 2009 CKD Epidemiology Collaboration formula [37].

Serum samples were collected from all participants following an overnight fast, then centrifuged at 3000 rpm for 10 min. Similarly, urine samples were processed by centrifugation at 1500 rpm for 10 min, and the supernatants were stored at −80°C for later analysis.

### 2.3. Laboratory Testing

Serum ATGL levels were quantified using an ELISA kit (CSB-E12688h, Cusabio, Houston, Texas), following the provided instructions. Similarly, serum TNFR-1 and urine KIM-1 were measured with ELISA kits (E-EL-H0217c, Elabscience, Houston, Texas; E-EL-H6029, Elabscience), according to the manufacturer's guidelines. The intra- and interassay coefficients of variation for all tests were kept below 10%.

Each experiment was performed in triplicate, and the average values were calculated to reduce measurement bias. For urine KIM-1, the results were standardized against urine Cr concentrations.

### 2.4. Statistical Methods

To evaluate the normality of the variable distributions, the Kolmogorov–Smirnov test and Q-Q plot were utilized. Continuous data were expressed as mean ± standard deviation (SD) when normally distributed, while median and interquartile ranges were used for nonnormally distributed data. Categorical variables were reported as frequencies and percentages. Significant differences between groups were assessed using one-way analysis of variance, Mann–Whitney *U* test, and chi-square tests for continuous and categorical data, respectively. Spearman's correlation analysis was conducted to examine relationships among different variables. The receiver operating characteristic (ROC) curve was utilized to evaluate the sensitivity and specificity of ATGL for detecting DKD. To investigate the link between serum ATGL levels and DKD, both univariate and multivariate logistic regression analyses were performed. Furthermore, restricted cubic splines (RCSs) were applied to capture the nonlinear relationship between serum ATGL and clinical parameters of DKD in both regression models. All statistical analyses were carried out using SPSS Statistics (Version 22.0; SPSS Inc., Chicago, Illinois, United States), R software (Version 4.2.2; http://www.R-project.org/), and MSTATA software (http://www.mstata.com). *p* values < 0.05 were considered statistically significant.

## 3. Results

### 3.1. Demographic and Baseline Information

A total of 236 participants were enrolled in this study, with their clinical characteristics summarized in Table 1. There were notable differences in various factors across the groups, including the proportion of males, BMI, duration of diabetes, smoking history, medical history of hypertension, hyperlipidemia, fatty liver, cardiovascular disease, and cerebrovascular disease. Additionally, significant disparities were observed in UACR, 24 h-UTP, renal function markers (eGFR, SCr, BUN, and UA), HCY, TG, HDL-C, and serum levels of ATGL, KIM-1/Cr, and TNFR-1. These variations align with the typical features of DKD. However, no significant differences were observed among the groups in terms of age, TC, or LDL-C levels.

**Figure 1:**
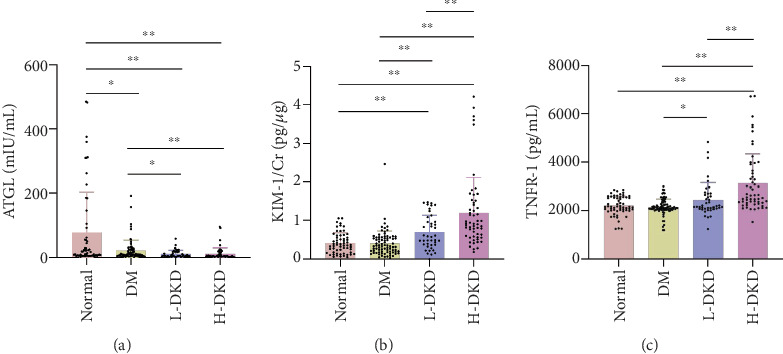
Contents of serum ATGL, urine KIM-1/Cr, and serum TNFR-1 in different groups. (a) The level of serum ATGL in HC group, DM group, L-DKD group, and H-DKD group. (b) The level of urinary KIM-1/Cr in HC group, DM group, L-DKD group, and H-DKD group. (c) The level of serum TNFR-1 in HC group, DM group, L-DKD group, and H-DKD group. Data were expressed as the mean ± SD. ⁣^∗^*p* < 0.05,^∗∗^*p* < 0.01.

As depicted in Figure 1a, serum ATGL levels were significantly lower in all three patient groups compared to healthy controls. When compared to the T2DM group, ATGL levels were also notably reduced in both the L-DKD and H-DKD groups. However, there was no notable difference in serum ATGL levels between the L-DKD and H-DKD groups.  Figure 1b illustrates that the KIM-1/Cr ratios were significantly elevated in both the L-DKD and H-DKD groups compared to the healthy control and T2DM groups, with the H-DKD group showing a notably higher ratio than the L-DKD group.  Figure 1c highlights that serum TNFR-1 levels were substantially higher in the H-DKD group compared to the other three groups.

### 3.2. Relationship of Serum ATGL Levels With Renal Function Parameters in T2DM Patients

**Figure 2:**
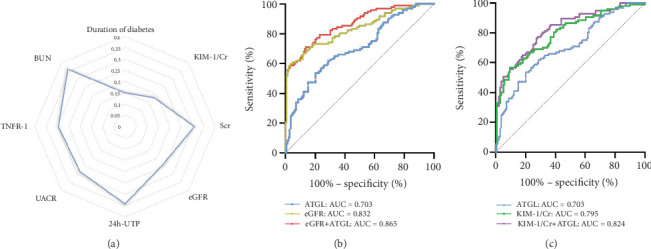
Correlation between serum ATGL and DKD parameters. (a) Correlation between serum ATGL- and DKD-related parameters (diabetes duration, UACR, 24 h-UTP, SCr, BUN, UA, TNFR-1, and KIM-1/Cr) based on Spearman's correlation test. (b) Sensitivity and specificity based on ROC curve (ATGL, eGFR, and eGFR + ATGL). (c) Sensitivity and specificity based on ROC curve (ATGL, KIM-1/Cr, and KIM-1/Cr + ATGL).

Spearman's analysis (Table 2, Figure 2a) revealed that ATGL level was inversely associated with diabetes duration (*R*^2^ = −0.150, *p* = 0.046), UACR (*R*^2^ = −0.283, *p* < 0.001), 24 h-UTP (*R*^2^ = −0.343, *p* < 0.001), SCr (*R*^2^ = −0.308, *p* < 0.001), BUN (*R*^2^ = −0.361, *p* < 0.001), UA (*R*^2^ = −0.238, *p* < 0.001), TNFR-1 (*R*^2^ = −0.296, *p* < 0.001), and KIM-1/Cr (*R*^2^ = −0.182, *p* = 0.005). On the other hand, ATGL levels showed a positive correlation with eGFR (*R*^2^ = 0.238, *p* < 0.001). Serum ATGL levels were significantly lower in individuals with a history of hypertension and hyperlipidemia. According to the ROC curve, ATGL demonstrated a diagnostic value for DKD patients (sensitivity = 0.536, specificity = 0.799, and Jordan index = 0.335). Sensitivity can be enhanced by comparing the combined diagnostic performance of multiple indicators with that of traditional clinical markers (eGFR: sensitivity = 0.598, specificity = 0.964, and Jordan index = 0.562; eGFR + ATGL: sensitivity = 0.711, specificity = 0.863, and Jordan index = 0.574; KIM-1/Cr: sensitivity = 0.567, specificity = 0.906, and Jordan index = 0.473; KIM-1/Cr + ATGL: sensitivity = 0.773, specificity = 0.719, and Jordan index = 0.492) (Figure 2b,c).

### 3.3. Clinical Characteristics of DKD at Different Serum ATGL Levels

**Figure 3:**
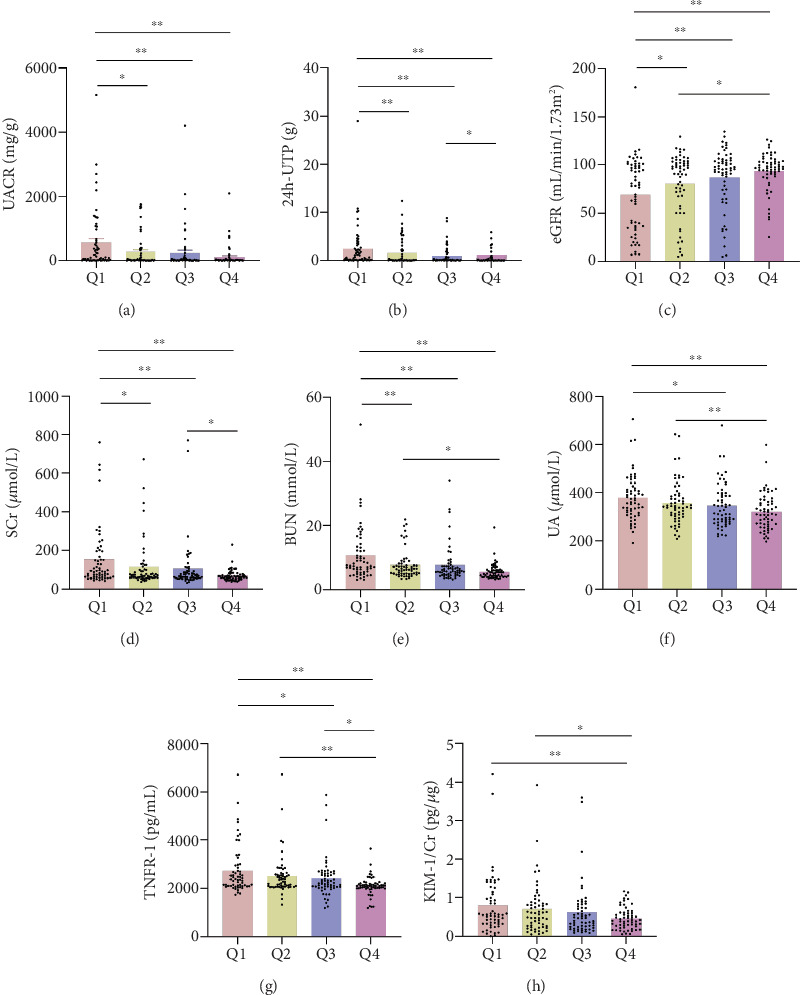
Expression differences of different DKD-related parameters in different serum ATGL quartiles. (a) UACR in different serum ATGL levels. (b) 24 h-UTP in different serum ATGL levels. (c) eGFR in different serum ATGL levels. (d) SCr in different serum ATGL levels. (e) BUN in different serum ATGL levels. (f) UA in different serum ATGL levels. (g) TNFR-1 in different serum ATGL levels. (h) KIM-1/Cr in different serum ATGL levels. Data were expressed as the mean ± SD. ⁣^∗^*p* < 0.05,^∗∗^*p* < 0.01.

In order to explore the association between serum ATGL levels and variables associated with DKD, participants were divided into four groups according to serum ATGL quartiles, as shown in Table 3. These groups were defined as follows: Q1 (serum ATGL ≤ 4.95 mIU/mL), Q2 (serum ATGL 4.95–8.20 mIU/mL), Q3 (serum ATGL 8.20–22.09 mIU/mL), and Q4 (serum ATGL ≥ 22.09 mIU/mL). No significant differences in sex, age, duration of diabetes, blood lipid levels (TG, LDL-C, and HDL-C), HCY, or KIM-1/Cr were found across the four groups. A higher prevalence of hypertension and hyperlipidemia was observed in the lower serum ATGL groups compared to those with higher ATGL levels (48 [81.36%] vs. 37 [62.71%] vs. 30 [50.85%] vs. 21 [35.59%]). As serum ATGL levels increased from the Q1 group, eGFR showed a gradual increase, while UACR, 24 h-UTP, SCr, BUN, UA, and TNFR-1 levels progressively decreased, with these differences being statistically significant. Further analysis revealed notable differences in the distribution of UACR, 24 h-UTP, eGFR, SCr, BUN, UA, and TNFR-1 across the various serum ATGL quartiles (Figure 3).

### 3.4. Serum ATGL and DKD: A Multivariate Logistic Regression Approach

**Figure 4:**
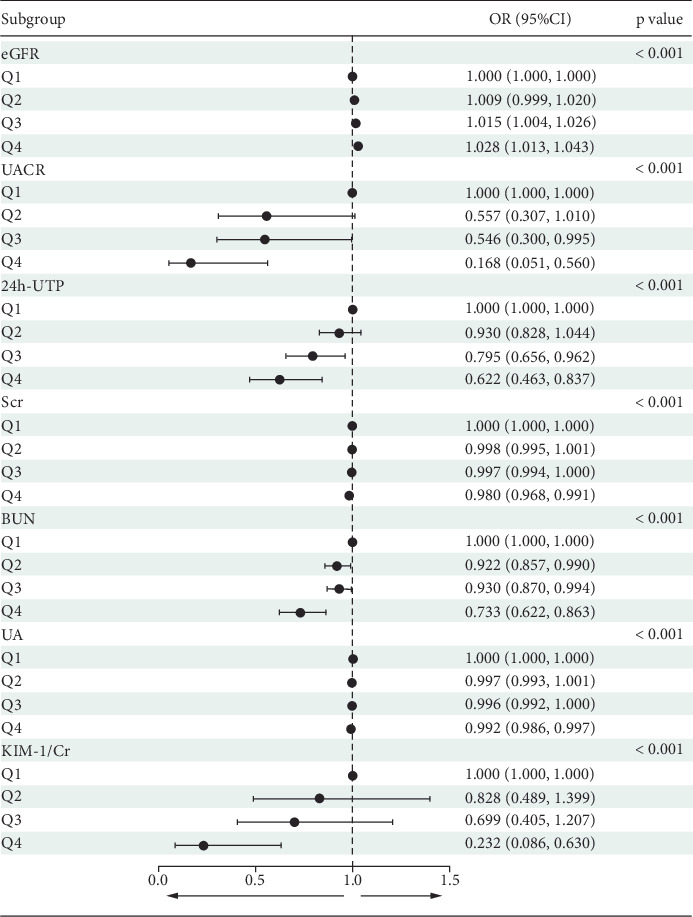
Forest plot of the relationship between quartiles of serum ATGL levels and DKD-related parameters based on logistic regression.

Participants were divided into four groups based on the quartiles of serum ATGL levels. Logistic regression analysis revealed that, with the Q1 group as the reference, the odds ratios (ORs) for elevated UACR, 24 h-UTP, SCr, BUN, UA, and KIM-1/Cr decreased progressively, while the ORs for higher eGFR increased as the serum ATGL quartile level rose. This finding suggests dose-dependent trends between serum ATGL and these key parameters such as eGFR, UACR, and 24 h-UTP (Figure 4). Furthermore, after adjusting for potential confounding variables, including sex, age, fatty liver, smoking history, TC, TG, LDL-C, HCY, UA, and TNFR-1, both univariate and multivariate logistic regression analyses confirmed that serum ATGL remained a significant protective factor against the development of DKD (Table 4).

### 3.5. Dose–Response Association Between Serum ATGL Levels and DKD Incidence

**Figure 5:**
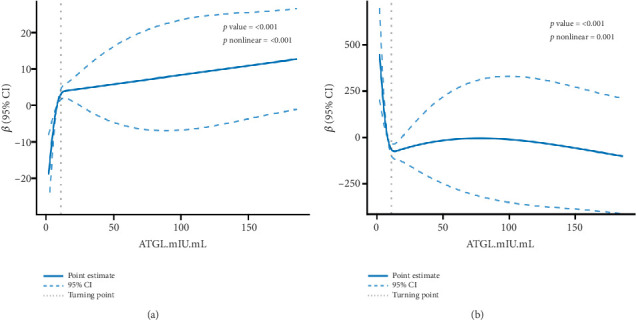
The restricted cubic spline (RCS) model fitting the association between serum ATGL and renal function. Adjustments were made for sex, age, fatty liver, and smoking. Solid lines represent multivariable adjusted *β*, and dashed lines represent 95% CIs obtained from restricted cubic spline regression. (a) Dose–response of serum ATGL and eGFR. (b) Dose–response of serum ATGL and UACR.

These results indicate a dose–response association of serum ATGL, eGFR, and UACR. Therefore, the RCS model was constructed to visualize the relationship among serum ATGL, eGFR, and UACR based on continuous changes in the independent variables. After accounting for confounders such as sex, age, history of fatty liver, and smoking history, there was a nonlinear relationship between serum ATGL and eGFR, as well as between ATGL and UACR (*p* < 0.001) (Figure 5). The inflection point was determined based on the RCS curve. The risk of UACR progression was reduced by 24.4% for each SD increase in serum ATGL, but the risk of UACR progression was reduced by 10.4% for each SD increase in serum ATGL when serum ATGL ≥ 11 mIU/mL. Similarly, for each SD increase in serum ATGL, the likelihood of an increase in eGFR increased by 36.3%, but for each SD increase in serum ATGL, the likelihood of an increase in eGFR increased by only 11.6% when serum ATGL ≥ 11 mIU/mL. These results suggested that serum ATGL is a stable protective factor against DKD (Table 5).

## 4. Discussion

This study examined the changes in serum ATGL levels in healthy controls, T2DM participants, and DKD participants. We first showed that serum ATGL levels were markedly reduced in DKD patients relative to both T2DM patients without DKD and healthy controls. Moreover, after accounting for potential confounding variables, a dose-dependent relationship between ATGL and renal function metrics was observed, with ATGL consistently acting as a protective factor against the development of DKD. Therefore, ATGL may have potential clinical value in delaying the occurrence of DKD in T2DM patients.

Thirty to forty percent of T2DM patients progress to DKD [38]. Therefore, the early prediction and identification of DKD are necessary. The development and progression of DKD are influenced by several factors, such as the duration of diabetes, smoking, serum UA, and hypertension [39–42]. Longer duration of DM increases the risk of DKD progression [43]. Serum UA can also promote the progression of DKD, and reducing serum UA levels can prevent or slow the decline in renal function [44]. DKD was strongly associated with hypertension (91.9% vs. 75.6%) and hyperlipidemia (86.6% vs. 78.2%) [45]. Renal outcomes tend to be worse when the mean systolic blood pressure is at least 140 mmHg and the mean diastolic blood pressure is at least 80 mmHg [46]. Our findings also revealed that these parameters were significantly elevated in the H-DKD group compared to the other three groups, aligning with previous research outcomes.

ATGL is a vital enzyme that regulates lipid metabolism. Lipid metabolism disorders can lead to kidney injury. Statins are first-line lipid-lowering drugs, and their long-term use can aggravate DKD through ELD in DM mice [47]. The overexpression of lipid receptors results in enhanced uptake of circulating lipids by the liver, leading to increased susceptibility to hepatic steatosis in patients with DM and dyslipidemia [48]. While ATGL is strongly linked to the onset and progression of fatty liver, this study revealed no substantial variations in the prevalence of fatty liver or lipid parameters across the ATGL quartiles. This may be due to the varying lipid-lowering treatments used by the participants [49].

ATGL plays an important role in tissues other than adipose tissue. ATGL deficiency or insufficient expression is associated with lipid accumulation in neutral lipid storage diseases, metabolic syndromes, and hepatic steatosis [50–52]. The decisive alteration in lipid homeostasis is not obesity but rather severe TG accumulation in nonfat tissues [53]. TG levels in adipose tissue were 2-fold higher in ATGL-deficient mice, 20-fold higher in myocardial tissue, 15-fold higher in kidney tissue, and 2-fold higher in the liver than in control mice [16]. Lipid metabolism is affected in ATGL-deficient mice and renal cells treated in vitro. ATGL-deficient mice show abnormal renal function and ectopic fat deposition in the kidneys [28, 29]. ATGL defects reduce the availability of FFAs, leading to increased glucose utilization, improved glucose tolerance, and enhanced insulin sensitivity [16]. In vitro inhibition of ATGL in human renal proximal tubular epithelial (HK-2) cells resulted in intracellular lipid accumulation, elevated levels of reactive oxygen species, and the initiation of apoptosis [29]. ATGL is widely expressed throughout the renal cortex, with a notable concentration on the apical or luminal surface of certain cortical tubules [54]. Research indicates that ATGL expression is reduced in the kidneys of DKD mice [55]. Moreover, kidney damage resulting from a high-fat diet (HFD) is also linked to reduced ATGL expression [56]. In line with these findings, our study revealed that serum ATGL concentrations were significantly reduced in DKD patients compared to individuals with T2DM and healthy controls.

Various factors regulate ATGL activity and expression. In the present study, DKD was more common in men than in women. Sex hormones are thought to be the primary factors contributing to the sex differences observed in DKD. However, whether there are gender differences in DKD is still controversial [57, 58]. Before menopause, nondiabetic women have a relative protection against kidney disease, but this protection diminishes after menopause and with the onset of diabetes [43]. Women with DM are at a higher risk of progressing to ESRD compared to their male counterparts with the same condition [59]. However, our study suggested no sex differences in serum ATGL levels, consistent with previous findings [60]. This study found that BMI increased with the progression of DM, indicating its influence on the onset and progression of DKD. A 17-year follow-up from the Swedish Diabetes Incidence Study revealed that BMI (*p* = 0.012) is a key predictor of DKD in patients aged 9–17 years [61]. Additionally, prior research has demonstrated that individuals who are overweight or obese tend to have lower serum ATGL levels compared to those in the nonobese group [20]. In our study, BMI gradually decreased with increasing ATGL levels across quartiles, suggesting that ATGL is related to body fat content. Serum ATGL levels and tissue ATGL expression are affected by several factors and are closely associated with renal lipid deposition. Under hypoxic conditions, the overexpression of hypoxia-inducible LD-associated protein in HK-2 cells and mouse kidney tissue with unilateral ureteral obstruction and unilateral ischemia–reperfusion injury downregulated ATGL. This leads to TG accumulation, causing defects in fatty acid oxidation [62]. After exposure to PM_2.5_, ELD and decreased ATGL expression were observed in the kidneys [55]. ATGL expression was also associated with increased Sirtuin 1 expression and forkhead Box O1 deacetylation [63]. Silencing of the Dff45-like effector C gene can increase ATGL expression, thereby promoting autophagy and inhibiting apoptosis to delay DKD progression in rats [64]. Among hemodialysis patients, the odds of wasting severity increased by 21% for each unit increase in serum ATGL concentration [65]. However, increasing ATGL expression in renal cells improved HFD-induced renal endocytosis defects [66]. Angiotensin 1–7 treatment increased renal ATGL expression, which reduces renal lipid accumulation in db/db mice [63]. Liraglutide was found to increase renal ATGL expression, which helped reduce the accumulation of ectopic lipids in the renal tubules of DKD rats, as well as mitigate palmitic acid–induced lipid buildup in renal tubular epithelial cells [27]. Our results also confirm that serum ATGL is a stable, independent protective factor against DKD.

The role of ATGL in protecting renal function in DKD patients remains unclear. Interestingly, ATGL-deficient mice showed an improvement in blood glucose levels, possibly due to the decreased TG degradation capacity of adipose tissue and reduced FFA release, leading to increased glucose breakdown to meet metabolic demands [16]. ATGL is a key molecular factor in both lipolysis and lipophagy. Ubiquitin-mediated degradation of ATGL inhibits FAO, thereby worsening renal LD accumulation and fibrosis, while *α*Klotho overexpression prevents ATGL ubiquitination to rescue FAO, thereby maintaining renal lipid homeostasis [67]. ATGL is an essential mediator of lipid ligand generation and is involved in the peroxisome proliferator–activated receptor (PPAR) activation. As nuclear receptors, PPARs are essential in controlling the expression of genes involved in energy metabolism and inflammatory processes [68]. ATGL deficiency reduces the mRNA expression of target genes regulated by PPAR-*α* and PPAR-*δ* [50]. Fenofibrate, an approved PPAR-*α* activator, can treat DKD by preventing lipid accumulation and apoptosis [69, 70]. Targeting ATGL as an intervention in renal lipid metabolism is expected to reduce renal injury.

Nevertheless, there are a few limitations to this study. Primarily, being cross-sectional in nature, it cannot determine whether the low serum ATGL levels are a cause or a consequence of DKD. The longitudinal association between ATGL and DKD requires further investigation, including follow-up. Secondly, this study did not assess the influence of factors such as diet, physical activity, and lipid-lowering medications on insulin resistance and lipid profiles, which may introduce some bias. Future research should thoroughly document and analyze these variables to offer more comprehensive and detailed insights. Thirdly, the findings of this study require further validation through research with larger sample sizes and more detailed subgroup analyses, with careful matching of significant sociodemographic variables. Lastly, the study population was relatively homogeneous in terms of ethnicity and geographic region, limiting the generalizability of the results. Thus, it is essential to examine the relationship between ATGL and DKD across diverse regions and ethnic groups for broader applicability.

## 5. Conclusion

In summary, this study is pioneering in demonstrating that serum ATGL levels are significantly reduced in DKD patients and are associated with disease development. Therefore, ATGL holds promise as a potential target for slowing the development of DKD. These results provide new perspectives on the molecular mechanisms contributing to renal injury in DKD patients with lipid metabolism disorders. Nonetheless, additional studies are required to investigate the long-term association between ATGL and DKD, along with its protective mechanisms.

## Figures and Tables

**Table 1:** Baseline demographic and clinical characteristics of the study population.

**Characteristics**	**Healthy control (** **n** = 59**)**	**DM (** **n** = 80**)**	**L-DKD (** **n** = 41**)**	**H-DKD (** **n** = 56**)**	**p** ** value**
Male (%)	27 (45.76)	38 (47.50)	27 (65.85)	37 (66.07)	0.036
Age (years)	56.39 ± 10.27	56.50 ± 10.10	58.10 ± 6.94	58.95 ± 10.46	0.407
Duration of diabetes (years)	/	8.12 ± 7.68	12.81 ± 7.77	13.75 ± 7.34	< 0.001
Hypertension (%)	7 (11.86)	45 (56.25)	31 (75.61)	53 (94.64)	< 0.001
Hyperlipidemia (%)	9 (15.25)	47 (58.75)	30 (73.17)	35 (62.50)	< 0.001
Fatty liver (%)	8 (13.56)	42 (52.50)	10 (24.39)	10 (17.86)	< 0.001
CVD (%)	7 (11.86)	28 (35.00)	13 (31.71)	24 (42.86)	0.002
Cerebrovascular disease (%)	1 (1.69)	16 (20.00)	9 (21.95)	17 (30.36)	0.001
Smokers (%)	5 (8.47)	18 (22.50)	10 (24.39)	20 (35.71)	0.006
BMI (kg/m^2^)	22.62 ± 3.55	24.90 ± 3.67	25.59 ± 4.39	29.56 ± 26.54	0.047
UACR (mg/g)	13.00 (4.97, 20.00)	3.36 (0.83, 11.90)	83.90 (50.00, 164.85)	947.31 (414.84, 1592.31)	< 0.001
24 h-UTP (g)	0.05 (0.02, 0.09)	0.09 (0.04, 0.13)	0.57 (0.28, 2.29)	3.57 (1.88, 5.31)	< 0.001
SCr (*μ*mol/L)	60.00 (52.50, 71.30)	61.60 (53.08, 73.90)	83.00 (63.00, 111.00)	158.55 (89.70, 274.25)	< 0.001
eGFR (mL/min/1.73 m^2^)	99.42 ± 12.35	98.70 ± 19.10	77.11 ± 27.99	45.94 ± 31.60	< 0.001
BUN (mmol/L)	5.15 ± 1.29	5.64 ± 1.70	8.12 ± 5.23	13.73 ± 8.24	< 0.001
UA (*μ*mol/L)	317.84 ± 74.91	333.75 ± 81.68	382.63 ± 105.69	386.87 ± 87.06	< 0.001
TC (mmol/L)	5.00 ± 1.33	4.69 ± 1.46	4.64 ± 1.19	5.06 ± 1.58	0.282
TG (mmol/L)	1.28 (0.87, 1.96)	1.42 (1.03, 2.24)	1.67 (1.25, 2.40)	1.74 (1.21, 2.35)	0.043
LDL-C (mmol/L)	2.95 ± 0.93	2.96 ± 1.06	2.86 ± 0.94	2.97 ± 1.08	0.955
HDL-C (mmol/L)	1.38 ± 0.36	1.21 ± 0.33	1.24 ± 0.36	1.26 ± 0.36	0.043
HCY (*μ*mol/L)	13.57 ± 5.98	10.85 ± 3.62	13.81 ± 5.80	19.15 ± 8.71	< 0.001
ATGL (mIU/mL)	13.94 (6.12, 61.27)	9.72 (5.42, 24.57)	5.42 (3.99, 18.19)	5.08 (2.75, 12.98)	< 0.001
TNFR-1 (pg/mL)	2200.84 ± 389.07	2138.87 ± 328.37	2439.65 ± 710.94	3120.98 ± 1223.58	< 0.001
KIM-1/Cr (pg/*μ*g)	0.41 ± 0.27	0.40 ± 0.32	0.69 ± 0.44	1.19 ± 0.94	< 0.001

Abbreviations: ATGL, adipose triglyceride lipase; BMI, body mass index; BUN, blood urea nitrogen; CVD, cardiovascular diseases; DKD, diabetic kidney disease; DM, diabetes mellitus; eGFR, estimated glomerular filtration rate; HCY, homocysteine; HDL-C, high-density lipoprotein cholesterol; KIM-1, kidney injury molecule-1; LDL-C, low-density lipoprotein cholesterol; SCr, serum creatinine; TC, total cholesterol; TG, triglycerides; TNFR-1, tumor necrosis factor receptor superfamily Member 1A; UA, uric acid; UACR, urinary albumin/creatinine ratio; UTP, urinary total protein.

**Table 2:** Association of serum ATGL with various patient characteristics.

**Characteristics**	**ATGL**	**p** ** value**
	**Quartile or ** **R** ** correlation factor**	
Sex		
Male (*n* = 129)	8.05 (4.90, 21.48)	0.231
Female (*n* = 107)	9.04 (5.04, 24.46)	
Age (years)	0.083	0.206
Duration of diabetes (years)	−0.150	0.046
Hypertension		
No (*n* = 100)	13.71 (6.12, 28.90)	< 0.001
Yes (*n* = 136)	5.77 (4.01, 17.31)	
Hyperlipidemia		
No (*n* = 115)	10.92 (5.37, 37.06)	< 0.001
Yes (*n* = 121)	6.02 (4.89, 17.84)	
Fatty liver		
No (*n* = 166)	8.14 (4.91, 21.91)	0.569
Yes (*n* = 70)	8.30 (5.12, 24.62)	
CVD		
No (*n* = 164)	8.14 (4.93, 23.02)	0.833
Yes (*n* = 72)	8.61 (4.98, 20.48)	
Cerebrovascular disease		
No (*n* = 193)	8.63 (4.96, 24.23)	0.099
Yes (*n* = 43)	6.41 (4.36, 18.67)	
Smokers		
No (*n* = 183)	8.16 (4.98, 24.02)	0.273
Yes (*n* = 53)	8.41 (3.64, 20.15)	
BMI (kg/m^2^)	−0.117	0.072
UACR (mg/g)	−0.283	< 0.001
24 h-UTP (g)	−0.343	< 0.001
SCr (*μ*mol/L)	−0.308	< 0.001
eGFR (mL/min/1.73 m^2^)	0.238	< 0.001
BUN (mmol/L)	−0.361	< 0.001
UA (*μ*mol/L)	−0.238	< 0.001
TC (mmol/L)	−0.062	0.341
TG (mmol/L)	−0.086	0.189
LDL-C (mmol/L)	−0.058	0.371
HDL-C (mmol/L)	−0.025	0.699
HCY (*μ*mol/L)	−0.078	0.233
ATGL (mIU/ml)	1	/
TNFR-1 (pg/mL)	−0.296	< 0.001
KIM-1/Cr (pg/*μ*g)	−0.182	0.005

Abbreviations: ATGL, adipose triglyceride lipase; BMI, body mass index; BUN, blood urea nitrogen; CVD, cardiovascular diseases; eGFR, estimated glomerular filtration rate; HCY, homocysteine; HDL-C, high-density lipoprotein cholesterol; KIM-1, kidney injury molecule-1; LDL-C, low-density lipoprotein cholesterol; SCr, serum creatinine; TC, total cholesterol; TG, triglycerides; TNFR-1, tumor necrosis factor receptor superfamily Member 1A; UA, uric acid; UACR, urinary albumin/creatinine ratio; UTP, urinary total protein.

**Table 3:** Based on the quartile of serum ATGL levels, participants' clinical characteristics, and biochemical parameters.

**Characteristics**	**Q1 (≤ 4.95)**	**Q2 (4.95–8.20)**	**Q3 (8.20–22.09)**	**Q4 (≥ 22.09)**	**p** ** value**
Male (%)	36 (61.02)	30 (50.85)	34 (57.63)	29 (49.15)	0.524
Age (years)	56.86 ± 10.28	57.17 ± 9.34	56.39 ± 10.00	58.90 ± 9.45	0.530
Duration of diabetes (years)	12.91 ± 7.69	9.48 ± 7.52	11.40 ± 8.10	9.33 ± 8.54	0.097
Hypertension (%)	48 (81.36)	37 (62.71)	30 (50.85)	21 (35.59)	< 0.001
Hyperlipidemia (%)	39 (66.10)	34 (57.63)	27 (45.76)	21 (35.59)	0.005
Fatty liver (%)	13 (22.03)	21 (35.59)	16 (27.12)	20 (33.90)	0.343
CVD (%)	16 (27.12)	18 (30.51)	21 (35.59)	17 (28.81)	0.772
Cerebrovascular disease (%)	13 (22.03)	14 (23.73)	9 (15.25)	7 (11.86)	0.293
Smokers (%)	18 (30.51)	7 (11.86)	18 (30.51)	10 (16.95)	0.026
BMI (kg/m^2^)	28.65 ± 26.13	24.75 ± 3.60	24.51 ± 3.33	24.33 ± 4.11	0.246
UACR (mg/g)	129.00 (10.98, 681.05)	19.70 (4.61, 329.46)	15.00 (3.38, 94.35)	15.00 (1.34, 22)	< 0.001
24 h-UTP (g)	1.04 (0.16, 3.22)	0.11 (0.04, 2.25)	0.13 (0.06, 0.77)	0.08 (0.03, 0.21)	< 0.001
SCr (*μ*mol/L)	89.60 (63.00, 194.50)	70.00 (58.70, 100.00)	65.90 (56.60, 91.40)	63.40 (52.20, 76.80)	< 0.001
eGFR (mL/min/1.73 m^2^)	69.46 ± 37.70	80.67 ± 32.07	86.76 ± 30.78	93.53 ± 19.61	< 0.001
BUN (mmol/L)	10.58 ± 8.13	7.70 ± 4.46	7.60 ± 5.63	5.60 ± 2.43	< 0.001
UA (*μ*mol/L)	378.97 ± 94.46	357.62 ± 90.20	345.98 ± 91.52	320.92 ± 76.47	0.005
TC (mmol/L)	4.41 (3.76, 5.68)	5.11 (4.31, 5.82)	4.45 (3.84, 5.17)	4.72 (4.12, 5.43)	0.047
TG (mmol/L)	1.60 (1.11, 2.26)	1.60 (1.27, 2.3)	1.41 (0.99, 2.065)	1.40 (0.92, 2.25)	0.527
LDL-C (mmol/L)	3.03 ± 1.22	3.04 ± 0.84	2.86 ± 1.07	2.84 ± 0.85	0.552
HDL-C (mmol/L)	1.27 ± 0.38	1.33 ± 0.40	1.23 ± 0.31	1.25 ± 0.31	0.436
HCY (*μ*mol/L)	15.96 ± 8.61	13.21 ± 5.82	13.48 ± 6.41	13.39 ± 5.84	0.090
ATGL (mIU/mL)	3.41 (2.28, 4.38)	5.57 (5.16, 6.46)	12.40 (9.75, 18.19)	49.39 (25.88, 104.50)	< 0.001
TNFR-1 (pg/mL)	2729.60 ± 992.55	2515.36 ± 821.94	2412.36 ± 823.45	2101.32 ± 387.53	< 0.001
KIM-1/Cr (pg/*μ*g)	0.55 (0.33, 1.14)	0.59 (0.27, 0.85)	0.40 (0.22, 0.76)	0.40 (0.25, 0.60)	0.055

Abbreviations: ATGL, adipose triglyceride lipase; BMI, body mass index; BUN, blood urea nitrogen; CVD, cardiovascular diseases; eGFR, estimated glomerular filtration rate; HCY, homocysteine; HDL-C, high-density lipoprotein cholesterol; KIM-1, kidney injury molecule-1; LDL-C, low-density lipoprotein cholesterol; SCr, serum creatinine; TC, total cholesterol; TG, triglycerides; TNFR-1, tumor necrosis factor receptor superfamily Member 1A; UA, uric acid; UACR, urinary albumin/creatinine ratio; UTP, urinary total protein.

**Table 4:** OR and 95% CI for having DKD in different ATGL levels based on logistic regression.

**ATGL (mIU/mL)**	**Crude**		**Model 1**		**Model 2**		**Model 3**	
	**OR (95% CI)**	**p** ** value**	**OR (95% CI)**	**p** ** value**	**OR (95% CI)**	**p** ** value**	**OR (95% CI)**	**p** ** value**
ATGL	0.977 (0.961, 0.993)	0.005	0.975 (0.959, 0.991)	0.002	0.973 (0.957, 0.989)	0.001	0.981 (0.966, 0.996)	0.015
Q1–Q2 (< 8.20)	0.623 (0.486, 0.798)	< 0.001	0.666 (0.511, 0.869)	0.003	0.648 (0.491, 0.855)	0.002	0.687 (0.509, 0.927)	0.014
Q3–Q4 (> 8.20)	0.988 (0.975, 1.000)	0.060	0.984 (0.971, 0.998)	0.025	0.984 (0.969, 0.998)	0.030	0.985 (0.970, 1.000)	0.044

*Note:* Model 1: adjusted for age, sex, fatty liver and smokers. Model 2: further adjusted for TC, TG, and LDL-C. Model 3: further adjusted for HCY, UA, and TNFR-1.

Abbreviations: ATGL, adipose triglyceride lipase; CI, confidence interval; DKD, diabetic kidney disease; HCY, homocysteine; LDL-C, low-density lipoprotein cholesterol; OR, odds ratio; TC, total cholesterol; TG, triglycerides; TNFR-1, tumor necrosis factor receptor superfamily Member 1A; UA, uric acid.

**Table 5:** Effect of standardized ATGL level on DKD adjusted coefficients from segmented linear regression analysis.

**Characteristic**	**ATGL (mIU/ml)**	**Beta per SD**	**OR (95% CI)**	**p** ** value**
UACR	< 11	−0.28	0.756 (0.644, 0.887)	< 0.001
	≥ 11	−0.11	0.896 (0.726, 1.094)	0.270
eGFR	< 11	0.31	1.363 (1.162, 1.600)	< 0.001
	≥ 11	0.11	1.116 (0.914, 1.363)	0.300

Abbreviations: ATGL, adipose triglyceride lipase; CI, confidence interval; DKD, diabetic kidney disease; eGFR, estimated glomerular filtration rate; SD, standard deviation; UACR, urinary albumin/creatinine ratio.

## Data Availability

The data supporting the findings of this study can be obtained from the corresponding authors upon reasonable request.
